# Educational Neuroscience Training for Teachers’ Technological Pedagogical Content Knowledge Construction

**DOI:** 10.3389/fpsyg.2021.792723

**Published:** 2021-12-24

**Authors:** Yulu Cui, Hai Zhang

**Affiliations:** ^1^School of Information Science and Technology, Northeast Normal University, Changchun, China; ^2^School of Media Science, Northeast Normal University, Changchun, China

**Keywords:** neuroscience, educational neuroscience, brain, teachers development, TPACK

## Abstract

The attention to the laws of the brain and the mechanism of learning in the smart education era becomes the starting point for the convergence and development of education and neuroscience, which also inspired educational neuroscience (EN) affecting the teacher’s development. Although teachers always have a general curiosity about EN and its applications, the limited knowledge hinders their general practice, neuromyths begin to emerge, and there is no evidence to directly show the connection between EN and teachers’ technological pedagogical content knowledge (TPACK) knowledge. Based on an EN teacher training program for 216 teachers, this study verifies that EN training programs can promote teachers’ understanding of EN-related knowledge, and EN is also correlated to teachers’ TPACK. However, the EN training program does not promote high well-being and satisfaction. The research also analyzes the process of teachers’ EN knowledge dissemination based on interviews, and the research conclusion can further reveal the necessity of EN training for teachers in the future.

## Introduction

In the 21st century, the development of information and communications technologies (ICTs) has promoted profound changes in education, and great changes have taken place in the teaching process and methods (Liu and Zhang, 2021). The construction of intelligent learning environment produced multimodal educational data, which provides great help in understanding the learner and teaching and even further promotes the professional development of teacher (Hai et al., 2020). The new learning technologies, tools, and platforms can provide for leaners to enhance and generate better experiences (Cui et al., 2019). Especially in the COVID-19 pandemic of 2020 and 2021, it is more important for teachers to use new technology and carry out professional development to improve the educational quality, promote the reform, and reconstruct education (Mo et al., 2021). However, it is obviously not easy for teachers to bridge the gap between theory and practice, especially in a scientific way (Zhang et al., 2020b).

In educational research and practice, evidence-based research and practice have gradually become the concern and discussion of teachers, school leaders, and educational researchers. In these discussions, topics about learning and the brain often appear in the smart education era. As more and more researchers begin to recognize the important role of the human brain and its mechanism in learning, the field of neuroscience has revealed more natural secrets about humans, especially the way we learn, leading to educational neuroscience (EN) gradually becoming one of the future directions of educational research. Thomas (2013) indicated that future educational practice can be changed by neuroscience, just like the contribution of science to medicine in history. In a report published by Welcome Trust, involving more than 1,000 teachers, more than 90% of these teachers said their understanding of neuroscience is affecting them and 80% of teachers said they would cooperate with neuroscientists on educational research (Simmonds, 2014). Although teachers have a general curiosity about EN and its applications, the limited knowledge hinders their general practice. Besides, professional practice based on EN is even more difficult even if they understand the mechanisms of the brain and learning, and this is obviously beyond their abilities (Simmonds, 2014).

Although EN is considered to be beneficial to teachers, some researchers still questioned if it is too far from the laboratories and classrooms. From the perspective of discipline logic, as an interdisciplinary research field, the construction of EN itself integrates the research paradigms of neuroscience, pedagogy, psychology, and other disciplines, and different paradigms promote the attention of EN to the learning process and cognitive law from the physiological level. But this is complex for teachers and leaders in the school. Neuroscience research aims to study the human brain and neural mechanism, which can be roughly divided into five levels: the gene and molecular genetic level, the neuronal activities and their links, the brain circuits and functional systems, the behavioral disorders and syndrome, and the human behaviors, where the first three levels are less applicable to education and are unlikely to affect the practical process of education and teaching (Devonshire and Dommett, 2010). A famous conclusion on EN and education is that the distance between neuroscience laboratory and classroom is a “bridge too far” by Bruer (1977). Besides, limited knowledge hinders teachers’ general practice, and lots of teachers do not know how EN occurs and affects their teaching, leading to neuromyth wide spreads. A survey of 583 teachers shows that although teachers are very interested in EN, no matter what subject or content basis, neuromyth is easy to produce (Rato et al., 2013). Researchers emphasize that when there is a lack of understanding of EN knowledge, it is very easy to be misled by neuromyth, especially in the implementation of teaching process without scientific judgment (Zhifeng, 2021). In China, this phenomenon is also common, for instance, many teachers recognize brain utilization rate, the critical period of brain learning, and the hemispheric advantage of the brain are of great value, which may lead them to inappropriate business training programs for teenagers.

Researchers emphasize that extensive teacher training programs should be promoted to increase teachers’ understanding of EN in practice (Dubinsky et al., 2013; Wilcox et al., 2020; Suresh et al., 2021). Although these views are considered to be helpful for teachers, there is still a lack of studies in training EN to promote teacher practice, or the knowledge construction in practice, which means not enough evidence is explored to verify the EN effectiveness in training EN programs for teachers. Besides, although it is well known that EN is useful to teachers, there is still not enough research to confirm whether EN can help teachers’ knowledge construction. Thus, in this research, researchers aimed at verifying the effectiveness of an EN training program for teachers based on the technological pedagogical content knowledge (TPACK) framework. Besides, the relationship between EN and TPACK elements is examined through questionnaires and interviews. Furthermore, the possible EN knowledge transmission path is further considered. These attempts help to understand whether teacher EN training program is effective and how to promote teachers’ knowledge construction in a smart education era to promote teacher professional development.

### Educational Neuroscience

Neuroscience is a science of the human nervous system. In early research, neuroscientists divided the brain according to the structural details of the brain and the working status of different regions, for example, dividing each hemisphere into four different regions and each region corresponds to different functions of cognition and learning, such as listening, memory, self-movement, and so on (Yanyun and Wenchao, 2011). Research on the brain prompts people to pay attention to the specific cognitive functions rather than analyzing the brain as a complex and whole part, leading to different research on reasoning, attention, memory, and reading. With the development of new technology, neuroscience provides a way to locate brain regions that are active in different processes or tasks, and these brand-new technics rely on the exploration of neurons: when neurons are active, their blood flow supply changes which in turn does help in tracking the active neurons in the brain (Abdullaev, 2014). The development of neuroscience helps to promote the basic understanding of the neural mechanism of the learning process, and this interdisciplinary research practice prompted EN to establish.

Educational neuroscience is related to neuroscience, psychology, and education. As an interdisciplinary research field, it also has different names, such as brain-based learning, cognitive neuropsychology, neuroeducation, etc. Researchers often use “educational neuroscience” because, in essence, it is to scientifically combine the findings of neuroscience research with the educational theory and practice to improve teaching and learning (Amran et al., 2019). Many studies conducted in this field are aimed at studying the brain because it is closely related to education. In this emerging research field, professionals, cognitive scientists, and neuroscientists collaborate to apply neuroscience research to change education (Nouri, 2016), and EN also better reflects a knowledge field centered on education, characterized by neuroscience and technology, and based on experiential, social, and biological evidence (Howard-Jones, 2011). The rise of EN stems from the change of neuroscience to education because anything that influences learning will eventually be based on the brain. Therefore, the understanding of how the brain works will affect educational practice and research. The demand for EN comes from two directions: neuroscientists emphasize that their work has the potential to improve education, and educators are keen to understand what neuroscience can provide for their practice (Howard-Jones, 2014). The establishment of the field of EN marks the arrival of an era that focuses on human brain learning models and mechanisms, which gives researchers, leaders, and teachers specific opportunities to reexamine educational practice and research.

Especially for teachers, it is necessary to review the teaching process under EN-related works. Researchers pointed out that if teachers know more about the brain of learners, the more they will be able to recognize the differences in many ways of teaching and learning (Amiel and Tan, 2019; Doukakis and Alexopoulos, 2020), and EN can help teachers understand the brain operating mechanism, promote teachers to change teaching strategies, and optimize teaching design, to implement better teaching activities (Sigman et al., 2014). Latest research indicated that it is very important to understand these physiological data because these cues generated from the intelligent learning environment, such as heart rate, respiration, and pupil, can be used to judge learners’ physical discomfort, fear, or reluctance to help better understand learning and teaching (Zhang et al., 2020c). Many studies have also confirmed the important role of EN for teachers, such as correctly dealing with obstacles and correcting mistakes in the learning process will help to connect nerves and promote the transformation of knowledge (Moser et al., 2011) and retrieving information with consistent patterns leads to stronger neural connections (Fernández and Morris, 2018). The learners will feel more enjoyable and stronger motivation to deal with the task when the learning task is in the zone of proximal development (Baars and Wijnia, 2018). In many cases, it is beneficial to teachers’ curriculum design and learning process and they can try to avoid asking mechanical, trivial, and non-challenging questions but put forward multi-dimensional methods to consider a concept or idea, and the neural pathways and learning results will also be strengthened in return (Doukakis and Alexopoulos, 2020). Besides, understanding the EN is proved valuable to focus on special learning disabilities and special learning groups, for instance, brain tracking technologies (fMRI, PET, MRI, and EEG) help find the differences in the brain structure of dyslexia children, such as reduced brain functional plasticity, insufficient neural network activation, etc., leading to good educational guidance and teaching intervention to alleviate children’s dyslexia and improve their attention (Qiang and Meiqi, 2021). The practice of EN can also help teachers pay attention to special learning groups such as ADHD and ASD, and these concerns help teachers design reasonable courses and effective methods in improving education and teaching (Churches et al., 2017). Studies also provide teachers with “unprecedented insight” into a formative assessment in real time and offer valuable information about students’ overall participation and teaching effect (Campbell, 2020).

The above research shows that it is of positive significance to consider learners and the learning process from the perspective of EN research. Teachers can rethink the design of teaching activities, use the developmental characteristics of brain evidence to promote participation, and create an ideal and harmonious classroom atmosphere with EN research conclusions. In a word, the emergence of EN for teachers reflects a research-based or evidence-centered teaching method (Tomlinson and Sousa, 2020), and the EN-based intervention may help to improve students’ self-efficacy, affect school performance, and have a positive impact on student’s progress in the future (Cherrier et al., 2020). This means that promoting the perceptions of EN knowledge may be conducive to teachers’ knowledge construction, especially for the understanding of teaching practice in the smart education era.

### Technological Pedagogical Content Knowledge

Theoretical construction is necessary for any research field, especially for the teachers’ knowledge construction and practice under a background of integrating technology in the 21st century. TPACK framework is proposed by Mishra and Koehler (2006), which contains three main components: content knowledge (CK), technology knowledge (TK), and pedagogy knowledge (PK), and the complex interactions between them in teacher knowledge. TPACK framework is regarded as a breakthrough in teacher knowledge research because it integrates technical knowledge into the traditional views of the integration of contents and pedagogy and forms an integrated knowledge framework with technology. Koehler and Mishra (2009) pointed that relationships between teaching and technology with technology due to social and contextual factors also complicate, and teachers are facing new challenges with technology. TPACK is considered as a theory of knowledge in action because it origins from pedagogical content knowledge (PCK) and the capabilities can be infused into teacher curriculum to support teaching and learning (Tondeur et al., 2017). It is greatly beneficial to teachers, especially preservice teachers, on account of the integration of technology, pedagogy, and content, which promotes the process of teaching practice (Tondeur et al., 2020). Nowadays, there are many studies on TPACK because it has brought beneficial enlightenments to teachers’ knowledge development, such as research in MOOC, STEM, Math, Chemistry, and all kinds of disciplines (Chong, 2021).

The increase of EN knowledge also affects teachers’ knowledge structure. Geake (2011) noted that understanding EN is many advantages, one of which is conducive to “new possibilities in pedagogy or curriculum design.” Gang (2020) also stressed that EN is conducive to the deep and further understanding of various educational elements such as instructional design and students evaluation process. Schwartz et al. (2019) indicated that EN “enriches pedagogical choices” and promoted much more student-centered pedagogies after a set of EN concepts courses. Improving teaching becomes an important dimension of EN application because the understanding of students’ brains is an effective way to improve the teaching process, which in turn contributes to better achievements for both teachers and students. Especially in some international investigations, for instance, BrainU courses have been implemented on a large scale, and these projects have effectively promoted continuous reflection on teachers’ teaching process and understanding of class practice (Dubinsky et al., 2019). The EN-related teacher programs have been promoted by many institutions over the past decades, such as the Royal Society in the United Kingdom, the International Society for Neuroscience, the Organisation for Economic Cooperation and Development, etc., and these projects are used to promote teachers’ understanding of EN and their teaching practice (Coch, 2018). Studies also stressed its benefits for empowering teachers’ abilities and promoting education reforms, especially during the COVID-19 pandemic period (Joseph and Thomas, 2021). These findings mean that teachers need to consider their practical skills and promote their literacy (Zhang et al., 2020a), especially technology-related skills under the perspective of EN to promote teaching effect in their instruction. Ching et al. (2020) also emphasized the necessity of forming a teacher’s neuroscience literacy and noted that an understanding of EN can also help against neuromyths. These understandings need to be conducted into operable teaching steps and to integrate related data-based decision-making literacy with subject content and teaching method knowledge (Mandinach and Schildkamp, 2021). In other words, in the whole complex process of taking instruction, teachers need not only TK, CK, and PK but also literacy and understanding related to the new technologies and EN-related works.

**FIGURE 1:**
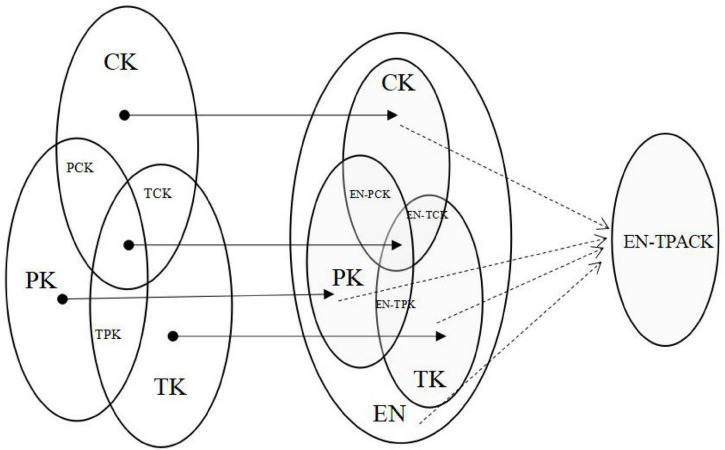
How EN influences TPACK and forms a new knowledge structure.

The existing studies paid much attention to teachers; however, not enough works were conducted on the role of EN knowledge in promoting teachers’ TPACK construction; considering the studies of EN and its benefits for teachers, we formed the idea that teachers’ TPACK may be formed based on EN. Due to the addition of a new element (EN), teachers’ knowledge structure also changes on the basis of EN-related concepts, works, and research, and a new composite knowledge framework may form by the integration of CK, TK, PK, and EN, as shown in Figure 1. Although EN knowledge is considered to be helpful to promote teachers’ knowledge, existing studies have failed to reveal the specific relationships between EN and various elements in the TPACK framework.

## Methodology

### Measures

**FIGURE 2:**
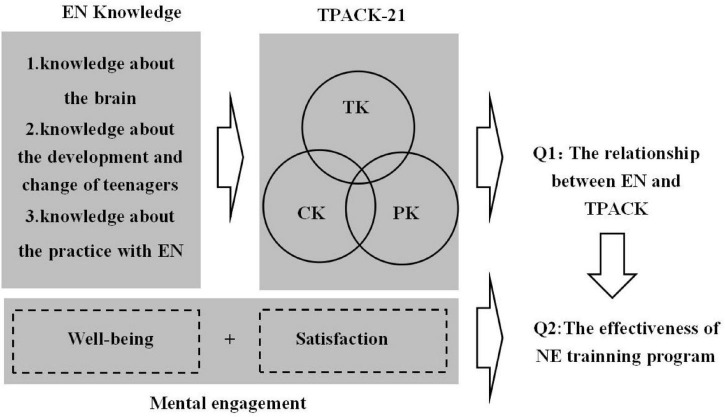
The research framework.

The understanding of EN cannot directly change teaching, but on the contrary, it will promote teachers’ understanding of learning, to implement better teaching (Jamaludin et al., 2019). Thus, in this study, the relationship between EN and teachers’ TPACK structure (Q1), and the effectiveness of the EN training program (Q2) through EN knowledge, TPACK-21, and mental engagement are explored. The research framework has been established to illustrate the possible impact of these three aspects as is shown in Figure 2:

Educational neuroscience: EN knowledge scale is measured from three aspects: knowledge about the brain (ENB, three items), knowledge about the development and change of teenagers (ENT, three items), and the knowledge about the practice with EN (ENP, three items). These three aspects were established according to the contents of the EN training program and were generated through discussions by researchers to ensure its reliability.

TPACK-21: TPACK scale employed TPACK-21 questionnaire, including CK (four items), PK (seven items), TK (four items), TCK (four items), PCK (six items), TPK (six items), and TPACK (seven items) developed by Valtonen et al. (2017). TPACK-21 was grounded on 21st-century skills, such as creativity, critical thinking, and problem solving. The questionnaire has been validated by CFA, and all statements have reached an adequate reliability level (α > 0.80).

Mental engagement: Mental engagement scale is considered from well-being (WB, seven items) and satisfaction (SA, six items), and each part has high reliability and validity according to Mo et al. (2021). These two aspects were considered because they are highly related to teacher training, especially satisfaction, and it is the best way to assess the effectiveness of the training program (Aimsrikul and Thitinaruemit, 2019).

### Procedure

The research constructs the initial questionnaire items through the Wenjuanxing platform^1^ and invites some teachers to fill in these questions. After the first investigation, researchers made appropriate adjustments to the questionnaire items according to the teachers’ feedback to better match the in-service participants. The survey was conducted after a teacher EN training program, lasting for a week. This program was designed for inspiring teachers with EN-related concepts, including content about the cognitive structure of the brain and the latest results of EN research on the classroom, such as why teenagers’ language sensitivity will become slow with age, why do teenagers writing skills improve little after middle school, and why teenagers always like to stay together and do things together. The content of the EN training program can be found in Appendix A, and the origin training content can be found in *My Teen Brain*, a teacher EN workshop implemented by Coleman (2021). After the program, teachers were invited to summarize the effects of this training and report remaining issues that will affect their understanding of EN in the program. Then, the interviewed data are further analyzed to conduct findings.

### Participants

The information of participants is sorted out after the questionnaire, where age, gender, working experience, educational background, EN experience before training (pre-EN), and EN training opportunity are considered. The participants contain 216 in-service teachers (men = 71, women = 145) in Jinlin Province, China. As is shown in Table 1, these teachers are recruited from the main subjects of elementary and secondary schools, for instance, Chinese, mathematics, English, etc. In terms of educational background, most of the teachers have a bachelor’s degree (*N* = 113, 52.3%) and master’s or above master’s degree (*N* = 71, 32.8%), with an average age = 34.2. Although these teachers have considerable teaching experience, for instance, most of the teachers have over 5 years of experience (68.1%), they responded that they had little understanding of EN before the training, ranking score from 1 to 2 on pre-EN experience, and only 3.2% teachers reported that they have a moderate understanding of EN. The feedback from participants shows that in-service teachers significantly lack opportunities for EN, and nearly 90% of teachers reported few opportunities to get appropriate EN training. This shows that the implementation of an EN training program for teachers is necessary.

**TABLE 1:** Profile of participants.

Variables	Category	Frequency	%	Variables	Category	Frequency	%
Age	20–29	67	31.1%	Educational background	Below bachelor’s	32	14.8%
	30–39	103	47.7%		Bachelor’s	113	52.3%
	40–49	34	15.7%		Master’s	65	30.1%
	>50	12	5.5%		Above master’s	6	2.8%
Working experience	1–5 years	69	31.9%	Pre-EN experience (1 = little, 5 = a lot)	1	162	75.0%
	5–10 years	89	41.2%		2	46	21.3%
	10–15 years	30	13.9%		3	7	3.2%
	15–20 years	18	8.3%		4	1	0.5%
	over 20 years	10	4.6%		5	0	0%
Gender	Female	145	67.1%	EN training opportunity (1 = little, 5 = a lot)	1	144	66.6%
					2	65	30.1%
	Male	71	32.9%		3	5	2.3%
					4	0	0%
					5	2	1.0%

## Results

### Data Analysis

In this work, SPSS 23.0 was used to examine in-service teachers’ EN knowledge, TPACK-21, and mental engagement after an EN training program.

First, the descriptive statistics and reliability analysis of each subscale are examined through SPSS, as shown in Table 2. From the measurement results of reliability statistics, the Cronbach’s alpha value of each subscale is over 0.80, and the total reliability result of 60 items is 0.933, indicating that the analyzed data have high internal consistency and strong reliability. The mean value of all subscales ranged from 2.656 to 4.195, where the ENB, ENT, and ENP only got a score from 2.656 to 3.003, showing that the teachers still have not enough understandings of the EN knowledge. Although teachers have limited EN-related knowledge, they also assigned relatively high scores to CK, PK, TK, PCK, TCK, TPK, and TPACK, ranging from 3.951 to 4.195. Besides, teachers reported WB and SA scores over 3.747, indicating that they got relative recognition of this EN training program, and their well-being and satisfaction are moderately accepted.

**TABLE 2:** Descriptive statistics and reliability of each subscale.

	N statistic	Minimum statistic	Maximum statistic	Mean statistic	Std. deviation statistic	Variance statistic	N of items	Cronbach’s α
ENB	216	1	5	3.003	0.897	0.805	3	0.891
ENT	216	1	5	2.960	0.862	0.743	3	0.910
ENP	216	1	5	2.656	0.869	0.755	3	0.867
CK	216	1	5	4.078	0.828	0.685	4	0.901
PK	216	1	5	4.104	0.825	0.681	7	0.917
TK	216	1	5	3.951	0.842	0.709	4	0.898
PCK	216	1	5	4.195	0.774	0.599	6	0.920
TCK	216	1	5	4.142	0.808	0.653	4	0.912
TPK	216	1	5	4.076	0.800	0.641	6	0.921
TPACK	216	1	5	4.087	0.805	0.649	7	0.927
WB	216	1	5	3.868	0.817	0.667	7	0.902
SA	216	1	5	3.747	0.762	0.58	6	0.900
Total							60	0.933

Second, the paired-samples test was used to explore the difference between pre-EN and the subscales of EN (ENB, ENT, and ENP). The data analysis results are as follows in Table 3, in which there are significant differences between the pre-EN and ENB (*p* = 0.00 < 0.05), there are significant differences between the pre-EN and ENT (*p* = 0.00 < 0.05), and there are significant differences between the pre-EN and ENT (*p* = 0.00 < 0.05), with a mean value of pre-EN, ENB, ENT, and ENP are 1.334, 3.003, 2.960, and 2.656, respectively. The results indicated that the three aspects of EN have increased varying degrees, and this EN training program can promote teachers’ understanding to a certain extent.

**TABLE 3:** Paired samples statistics of pre-EN and the subscales of EN.

		Mean	Paired differences					t	df	Sig. (two-tailed)
			Mean	Std. deviation	Std. Error Mean	95% Confidence interval of the difference				
						Lower	Upper			
Pair 1	Pre-EN and ENB	1.334 3.003	1.667	1.424	0.097	0.338	0.720	5.463	215	0.000
Pair 2	Pre-EN and ENT	1.334 2.960	1.626	1.456	0.099	0.377	0.768	5.781	215	0.000
Pair 3	Pre-EN and ENP	1.334 2.656	1.322	1.424	0.097	0.685	1.067	9.044	215	0.000

Third, the Pearson correlation analysis was used to test the correlations between the subscales of EN, TPACK-21, and mental engagement. As shown in Table 4, the correlation analysis results showed that there were high correlations between the subscales of EN and TPACK, for instance, the correlation between ENB and PCK was 0.822 (*p* < 0.05). However, the correlation between the subscales of EN and mental engagement and the correlation between the subscales of TPACK-21 and mental engagement were not supported. Besides, the subscales of mental engagement had internal consistency, where the correlation between well-being (WB) and satisfaction (SA) was 0.872 (*p* < 0.05). This shows that the cognition of EN knowledge will affect the dimensions of teachers’ TPACK structure, and TPACK also has connections in its complex framework. Although teacher training is designed for EN knowledge, the program does not obviously increase teachers’ satisfaction and well-being.

**TABLE 4:** Correlations between EN, TPACK-21, and mental engagement.

		ENT	ENP	ENB	CK	PK	TK	PCK	TCK	TPK	TPACK	WB	SA
ENT	Pearson correlation	1	0.929**	0.821**	0.807**	0.750**	0.678**	0.723**	0.712**	0.657**	0.696**	–0.012	–0.019
	Sig. (two-tailed)		0.000	0.000	0.000	0.000	0.000	0.000	0.000	0.000	0.000	0.860	0.784
ENP	Pearson correlation	0.929**	1	0.911**	0.882**	0.825**	0.753**	0.797**	0.776**	0.730**	0.751**	0.020	0.021
	Sig. (two -tailed)	0.000		0.000	0.000	0.000	0.000	0.000	0.000	0.000	0.000	0.767	0.761
ENB	Pearson correlation	0.821**	0.911**	1	0.877**	0.821**	0.729**	0.822**	0.784**	0.737**	0.747**	0.011	0.026
	Sig. (two -tailed)	0.000	0.000		0.000	0.000	0.000	0.000	0.000	0.000	0.000	0.877	0.709
CK	Pearson correlation	0.807**	0.882**	0.877**	1	0.938**	0.807**	0.888**	0.856**	0.805**	0.827**	0.000	0.025
	Sig. (two-tailed)	0.000	0.000	0.000		0.000	0.000	0.000	0.000	0.000	0.000	1.000	0.716
PK	Pearson correlation	0.750**	0.825**	0.821**	0.938**	1	0.852**	0.885**	0.860**	0.816**	0.840**	–0.025	0.012
	Sig. (two-tailed)	0.000	0.000	0.000	0.000		0.000	0.000	0.000	0.000	0.000	0.716	0.860
TK	Pearson correlation	0.678**	0.753**	0.729**	0.807**	0.852**	1	0.804**	0.816**	0.854**	0.834**	0.019	0.054
	Sig. (two-tailed)	0.000	0.000	0.000	0.000	0.000		0.000	0.000	0.000	0.000	0.786	0.427
PCK	Pearson correlation	0.723**	0.797**	0.822**	0.888**	0.885**	0.804**	1	0.935**	0.890**	0.889**	–0.011	0.023
	Sig. (two-tailed)	0.000	0.000	0.000	0.000	0.000	0.000		0.000	0.000	0.000	0.870	0.740
TCK	Pearson correlation	0.712**	0.776**	0.784**	0.856**	0.860**	0.816**	0.935**	1	0.899**	0.916**	–0.002	0.031
	Sig. (two-tailed)	0.000	0.000	0.000	0.000	0.000	0.000	0.000		0.000	0.000	0.976	0.651
TPK	Pearson correlation	0.657**	0.730**	0.737**	0.805**	0.816**	0.854**	.890**	0.899**	1	0.940**	0.052	0.062
	Sig. (two-tailed)	0.000	0.000	0.000	0.000	0.000	0.000	0.000	0.000		0.000	0.449	0.365
TPACK	Pearson correlation	0.696**	0.751**	0.747**	0.827**	0.840**	0.834**	0.889**	0.916**	0.940**	1	0.038	0.046
	Sig. (two-tailed)	0.000	0.000	0.000	0.000	0.000	0.000	0.000	0.000	0.000		0.582	0.501
WB	Pearson correlation	–0.012	0.020	0.011	0.000	–0.025	0.019	–0.011	–0.002	0.052	0.038	1	0.872**
	Sig. (two-tailed)	0.860	0.767	0.877	1.000	0.716	0.786	0.870	0.976	0.449	0.582		0.000
SA	Pearson correlation	–0.019	0.021	0.026	0.025	0.012	0.054	0.023	0.031	0.062	0.046	0.872**	1
	Sig. (two-tailed)	0.784	0.761	0.709	0.716	0.860	0.427	0.740	0.651	0.365	0.501	0.000	

**Correlation is significant at the 0.01 level (two-tailed).

### Interview Analysis

Through the above analysis, it is found that although EN knowledge was significantly correlated with TPACK, it did not affect the satisfaction and well-being of the training program. Although teachers’ EN knowledge increased significantly, it did not reach a very high level when compared with TPACK. After the program, 10% of teachers (T1–T22) were invited to summarize the results of this training, and the interviewed data were further analyzed. First, it was found that teachers showed great interest in the EN training, and teacher T6 concludes that “learning something about the brain are very novel comparing with traditional training programs,”, and T10 also stressed that the curriculum and key points are quite different from “technology, teaching method training projects.” This knowledge related to teenagers’ brains helps them recognize the difference between teenagers and adults, which “helps them reconsider teaching design process” as described by teachers T4, T6, and T13. Second, it is found that there are still difficulties in understanding the knowledge of EN although they have received related training, especially for the knowledge in practice (ENP). Teacher 3 said, “Some descriptions are too vague for me to understand.” “Although the teacher explained many theories about neuroscience, he didn’t tell us how to do the best in practice,” which was agreed by T3, T7, and T9. A detailed matrix data analysis chart was formed to better interpret the views of the teachers, as shown in Table 5.

**TABLE 5:** Views about learning EN in the teacher training program.

Views		T1	T2	T3	T4	T5	T6	T7	T8	T9	T10	T11	T12	T13	T14	T15	T16	T17	T18	T19	T20	T21	T22
Learning about EN is novel compared with traditional training programs.	EN is a new field for me.	*		*	*	*	*	*	*	*	*	*	*	*		*	*		*	*	*	*	*
	EN is a new knowledge for me.	*		*	*	*	*		*		*	*	*	*		*	*	*	*	*	*	*	*
	EN is a new teaching method for me.	*	*	*	*		*	*	*	*		*	*	*	*	*	*	*	*	*	*		*
Learning about EN helps re-consider the teaching design process.	EN helps understanding student.	*			*	*	*	*	*	*	*	*	*	*	*	*	*	*			*	*	*
	EN helps understanding resources.		*				*		*						*			*				*	
	EN helps understanding teaching.	*	*	*	*		*	*	*	*			*	*	*		*	*	*	*	*	*	*
Learning about EN still meets difficulties.	EN is too vague for me to understanding in theory.	*	*	*		*	*			*	*		*	*	*	*	*		*	*	*	*	*
	EN is too difficult for me to understanding in practice.			*	*	*		*	*	*		*	*					*	*	*			
	I rarely have chance to communicate with EN experts		*				*		*					*	*		*	*	*			*	*

The table was organized according to the interviews, and “*” means teachers agree with this view during the interview.

The findings of Table 5 indicate that EN is a new field for most of the teachers, and how to explain the views of EN to teachers may be more important than EN itself. It is not easy to construct the “bridge” between science research and classrooms. This may require neuroscientists and educators to establish a common discourse to promote better training effects. Through further discussions, we discussed with teachers the possible paths of EN knowledge dissemination, such as obtaining difficulties, media reasons, etc. This part will be described in detail in the discussion.

## Discussion

In this research, we conducted a training program on EN knowledge for in-service teachers and verified the relationship between EN and TPACK, which indicates that promoting teachers’ understanding of EN may be conducive to teachers’ knowledge construction. However, it is also found that the effect of the EN training program is limited, and the improvement of teachers’ EN-related knowledge, such as the knowledge about the brain (ENB), has not reached a very ideal level, and only moderate well-being and satisfaction are stressed. After analysis with SPSS and interview, this study gets the following conclusions.

First, a necessary training program can promote teachers’ understanding of EN. In this research, teachers’ understandings of EN knowledge have generally improved, especially for ENB, ENT, and ENP. Although they did not reach the level of 4/5, and the highest was only 3.003, it has made a lot of progress compared with the mean score of 1.334 of the EN before they get trained (pre-EN). This confirms the views that promoting teacher training can increase teachers’ cognitions in the field of neuroscience (Roehrig et al., 2012; Dubinsky et al., 2013). Since EN is a field related to education, many experts emphasize that the necessary teacher training is an effective way to connect neuroscience and education. The professional knowledge of EN is taught to teachers instead of directly promoting the effect of education practice, which may promote teachers’ teaching beliefs and lead to better instruction (Gutshall, 2020). Researchers also indicated that it is necessary to build a bridge between research and educational practice by forming a partnership (MacMahon et al., 2021), and it is also important to reciprocate interactions for neuroscience and education (Aronsson, 2020).

Second, it was found that EN knowledge is highly related to TPACK (*p* < 0.05) and can promote the development of teachers’ TPACK to a certain extent. Previous studies stressed that EN does a great help in inspiring teachers to develop brain-based learning and encourages students to learn independently (Amelia et al., 2021). Similar to these views, some teachers in this work reported that they can rethink the instructional design process from the research of neuroscience and the brain. Educational neuroscience knowledge helps them better integrate technology (TK), pedagogy (PK), and subject content knowledge (CK), which means that EN may promote teachers to form a new TPACK structure, for instance, EN-TPACK mentioned in Figure 1. Although the general well-being and satisfaction of teachers in this study are not very high, which can only reach 3.868 and 3.747, the interview with teachers indicates that the teachers’ understanding of EN has increased significantly and can further increase their reflection on teaching, as described by teacher T4, T6, and T13. This is consistent with the research of Elouafi et al. (2021) because it can help teachers better carry out instructional design, question and renew all practices, for instance, course content, sequences, rhythm, execution, and evaluation.

**FIGURE 3:**
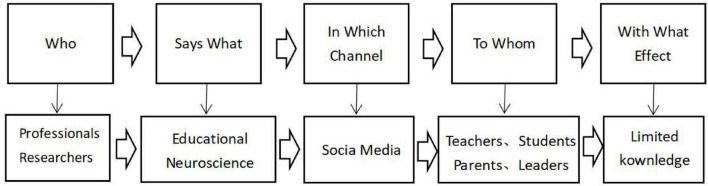
EN knowledge dissemination based on Lasswell’s 5W model.

Finally, there are still many difficulties in EN knowledge dissemination, although teachers get specifically trained. Regardless of the regional development levels and teaching levels, neuromyth will produce and affect the practical process of education, and a survey of 583 teachers in Portugal verified this conclusion, conducted by Rato et al. (2013). In this study, teachers pointed that they encountered many difficulties, such as vague knowledge and difficulty in practice, which shows that the causes of limited EN knowledge are complex. Lasswell’s 5W model (Wenxiu, 2015) inspired us how the dissemination of EN produces neuromyths and hinders teachers’ knowledge development, as shown in Figure 3. From the perspective of communication mechanism, limited EN knowledge of teachers came initially from different targets and discipline backgrounds. Neuroscience studies the brain and physiological mechanism, for instance, the genes, neuronal activities, and their links, and lots of research cannot be directly applied to change education (Devonshire and Dommett, 2010). Researches also points out that neuroscience and education lack common discourse, especially professional terms, vocabulary, literature, research and methods, which makes the concept and knowledge too vague to directly guide practice (Qiang and Meiqi, 2021). What’s more, the unscientific interpretations (oversimplified or over-interpreted) by the mass social media often make the conclusions too blurred to use for improving teaching from a scientific perspective (Dekker et al., 2012). Most importantly, the focus of EN training should be to teach teachers how to change the teaching process in practice, not to introduce relevant knowledge in theory. This means that in teacher EN training programs, we need to re-interpret and re-explain the relatively scientific professional knowledge in a way that teachers can easily understand to guide learning and instructing. This work requires the joint efforts of experts, scholars, and teachers to change practice in classrooms.

## Conclusion

Educational neuroscience is a systematic science to understand the occurrence law of human brain learning and may help teachers understand the general laws and characteristics of teaching, to change teachers’ traditional teaching methods. Although teachers expect to use the research results of EN to change the teaching process, it is undeniable that teachers’ practical level and understanding of EN are still limited compared with the content, psychology, and technical knowledge. In many cases, the teacher knowledge related to EN is on the edge of knowledge structure, and most of the teachers are found to lack an EN background or curriculum experience (Amran and Bakar, 2020). Therefore, it is necessary to promote specific EN training programs for teachers. The viewpoints of EN should be recommended and added to the pre-and in-service teachers’ training courses and programs to promote better teaching.

The original idea of this study is derived from the BrainU project developed by Dubinsky et al. (2013). The project aims to promote teachers’ educational practice by teaching teachers related concepts of neuroscience. Through a period of workshop projects, a team of expert teachers can be established to integrate the concepts, activities, demonstrations, and experiments of neuroscience into the classroom, and increase teachers’ use of inquiry teaching methods (Roehrig et al., 2012). Our research further verifies that it is of positive significance to teachers’ training EN-related knowledge, which is also helpful for the construction of teachers’ TPACK construction. The knowledge about ENB, ENT, and ENP is examined, and high correlations with the subscales of TPACK were found. Researchers also proposed that teachers should get trained according to the TPACK and EN to promote planning, design, and teaching practice (Doukakis et al., 2021), and this indicated that the understanding of EN can help teachers further master technology, subject content, and pedagogy in practice, so as to better carry out daily teaching. Thus, a new composite knowledge framework EN-TPACK is recommended to be established for both pre and in-service teachers to enhance their teaching practice. But at the same time, the study failed to reach the most ideal state. Although teachers’ knowledge of EN has increased, their sense of well-being and satisfaction cannot report a high level. In further research, this research would reduce the possible obstacles to teachers’ knowledge acquisition and dissemination, so as to promote teachers’ engagement and practice.

## Limitations and Future Research

This research enlightens teachers and increases their EN knowledge by the training program under EN. It is also helpful to further review the technology, pedagogy, and content knowledge, promoting the development of teachers’ TPACK. Future research needs to expand more samples and adopt a cyclic and iterative approach to verify the necessity of EN training for teachers, school managers, and even parents. Empirical research, for instance, comparative experiments for EN, is also recommended to evaluate teachers’ learning outcomes in authentic learning environments. In addition, although the analysis of the EN dissemination process may be inadequate, it will help to understand the generation path of teacher knowledge in practice, thereby promoting this emerging field of EN. Future research should also focus on reducing the obstacles of knowledge dissemination to promote teachers’ understanding of EN, TPACK, and its implementation in practice.

## Data Availability Statement

The original contributions presented in the study are included in the article/supplementary material, further inquiries can be directed to the corresponding author/s.

## Author Contributions

YC carried out the whole formal analysis, investigation, and writing—original draft. HZ edited the original manuscript to ensure that it reaches the standard of publishing, carried out project administration, and contributed to supervision and resources. Both authors contributed to the article and approved the submitted version.

## Conflict of Interest

The authors declare that the research was conducted in the absence of any commercial or financial relationships that could be construed as a potential conflict of interest.

## Publisher’s Note

All claims expressed in this article are solely those of the authors and do not necessarily represent those of their affiliated organizations, or those of the publisher, the editors and the reviewers. Any product that may be evaluated in this article, or claim that may be made by its manufacturer, is not guaranteed or endorsed by the publisher.

## Footnotes

1
https://www.wjx.cn/

## Appendix A

### The Content of the Educational Neuroscience Training Program for Teachers

Stage 1. Begin

✓ Elaborate the background of the educational neuroscience training program.
✓ Neuromyths we often encountered.
✓ Changing teachers’ views of the teenage brain.
✓ Stress the importance of educational neuroscience knowledge.

Stage 1. Key Points

✓ The key changes of the brain and teenagers’ transformation (development of various parts of the brain and the importance of pruning and hormone variation).
✓ Learning, classroom performance, and executive function (types of learning, changes in the brain as a result of learning, the meaning of executive function, and how this links with classroom performance).
✓ Hormones and reward processing of teenagers (how hormone levels vary and why rewards are especially important for teenagers).
✓ The social brain of teenagers (why the peer group matters).
✓ Sleep and healthy (why teenagers need sufficient sleep, and how sleep affects healthy).

Stage 3. Solutions

✓ For teachers (what teachers need to do under educational neuroscience).
✓ For parents (what parents need to do under educational neuroscience).
✓ For school managers (what school managers need to do under educational neuroscience).

Stage 4. Discussion

✓ What do you think the role of educational neuroscience?
✓ Can educational neuroscience help you in practice or knowledge development?
✓ Whether there are remaining issues or difficulties affecting your understandings and practice in class with educational neuroscience?
